# Understanding cachexia as a cancer metabolism syndrome

**DOI:** 10.1038/oncsis.2016.3

**Published:** 2016-02-22

**Authors:** P E Porporato

**Affiliations:** 1 Pole of Pharmacology, Institut de Recherche Expérimentale et Clinique (IREC), Université catholique de Louvain (UCL), Brussels, Belgium

## Abstract

Metabolic reprogramming occurs in tumors to foster cancer cell proliferation, survival and metastasis, but as well at a systemic level affecting the whole organism, eventually leading to cancer cachexia. Indeed, as cancer cells rely on external sources of nitrogen and carbon skeleton to grow, systemic metabolic deregulation promoting tissue wasting and metabolites mobilization ultimately supports tumor growth. Cachectic patients experience a wide range of symptoms affecting several organ functions such as muscle, liver, brain, immune system and heart, collectively decreasing patients' quality of life and worsening their prognosis. Moreover, cachexia is estimated to be the direct cause of at least 20% of cancer deaths. The main aspect of cachexia syndrome is the unstoppable skeletal muscle and fat storage wasting, even with an adequate caloric intake, resulting in nutrient mobilization – both directly as lipid and amino acids and indirectly as glucose derived from the exploitation of liver gluconeogenesis – that reaches the tumor through the bloodstream. From a metabolic standpoint, cachectic host develops a wide range of dysfunctions, from increased insulin and IGF-1 resistance to induction of mitochondrial uncoupling proteins and fat tissue browning resulting in an increased energy expenditure and heat generation, even at rest. For a long time, cachexia has been merely considered an epiphenomenon of end-stage tumors. However, in specific tumor types, such as pancreatic cancers, it is now clear that patients present markers of tissue wasting at a stage in which tumor is not yet clinically detectable, and that host amino acid supply is required for tumor growth. Indeed, tumor cells actively promote tissue wasting by secreting specific factors such as parathyroid hormone-related protein and micro RNAs. Understanding the molecular and metabolic mediators of cachexia will not only advance therapeutic approaches against cancer, but also improve patients' quality of life.

## Introduction

Cachexia is a life-threatening condition associated with several pathologies.^1^ It is particularly relevant in cancer patients, where it occurs in up to 80% of cancers. Cachexia is a marker of unfavorable prognosis, it affects the majority of patients with advanced cancer^2^ and it represents the direct cause of at least 20% of cancer-associated deaths.^1^

As it affects multiple organs, cachexia is an extremely complex disease, which severity is difficult to assess objectively. Indeed, only recently a method for staging cachexia extent has been proposed and validated.^3^

Typical symptoms of cachexia are massive loss of total body mass, anorexia, general inflammation and pronounced muscle-wasting resulting in a drastic decrease of quality of life.^4^ Furthermore, as broad muscular wasting also involves chest, diaphragm and cardiac muscle, it is not surprising that the majority of cancer deaths are related to respiratory^5^ or cardiac failure.^6^

Aside from being a direct cause of cancer death, cachexia also limits the therapeutic options as cachectic patients are normally less tolerant to radio- and chemotherapy because of general weakness and discomfort.^7^ Moreover, cachectic patients present a reduced response to therapy.^8^

Despite one of the main feature of cachexia being anorexia, the mode of tissue wasting is completely different from the one induced by starvation.^9^ Of note, treating anorexia through parenteral nutrition does not reverse cachexia, indicating that the decreased calories intake is not the primary cause of the disease.^9^

Unlike starvation, which primarily affects fat tissue, skeletal muscle is the major target of wasting in cachectic patients, suggesting a different signaling pathway targeting muscle loss.^10^ However, even though the main tissue affected by cachexia is the skeletal muscle, cachexia cannot be reduced to a muscle-wasting syndrome. Indeed, several other organs such as liver, heart, fat tissue and brain are affected, making cachexia a true multi-organ syndrome.^11^

To provide a clear definition of the molecular and metabolic determinants of tissue wasting, it is vital to apply a systemic approach in defining the contribution of each single organ to the cachectic process and to understand the role of tumor in this process and the interplay between the two compartments.

Indeed, although cachexia is a metabolic disorder characterized by tissue wasting, resistance to anabolic signals and an overall catabolic state, cancers, on the other side are highly proliferating and energy-demanding tissues.^12^ Consequently, the metabolic alterations present in cachectic patients results in a negative energy balance and into the release of nutrients in the bloodstream, further supporting tumor growth.^11^

Therefore, it is important to investigate the interplay between these two compartments and to understand how cancers promote this pathologic state to foster its own progression.

### Immune system

Inflammation is a double-edged sword in cancer. Aside from the natural role of immune system in controlling tumor growth, ultimate cancer cells hijack the immune system to produce specific cytokines promoting tumor growth, survival and progression.^13^ Chronic inflammation is also a major driver of cachexia (Table 1), as it affects the function of several tissues such as skeletal muscle, fat, brain and liver.^14^ Indeed, several pro-inflammatory cytokines promote cachexia: tumor necrosis factor alpha (TNFα), interleukin-6 and -1 (IL-6/IL-1) and interferon gamma.^15^

TNFα, initially named cachectin,^16^ is probably the most characterized cytokine in cachexia as it promotes anorexia^17^ and skeletal muscle wasting mainly through the NF-kB pathway.^18^ TNFα blockade (etanercept) provided promising results in improving cachexia-associated fatigue in a small cohort of cancer patients.^19^ However, recent trials using neutralizing antibodies against TNFα showed no benefit, suggesting that targeting TNFα alone is not sufficient to prevent cachexia.^20^

TNFα can also synergize with interferon gamma^21^ and IL-1^22^ in promoting muscle wasting. Despite that IL-1 itself promotes anorexia^23^ using IL-1 receptor antagonist was not sufficient to impair cachexia progression in a rat model.^24^ Nevertheless, human polymorphisms in IL-1B gene, resulting in augmented levels of IL-1β, were associated with a negative prognostic value,^25^ indicating the involvement of IL-1 pro-inflammatory cytokines in the pathogenesis of cachexia. A study in cancer patients identified increased circulating levels of cytokines (IL-1α, IL-6 and TNFα), suggesting the presence of a robust network of cytokines collectively promoting cachexia.^15^ IL-6 can directly drive cachexia in specific murine models^26^ and acute phase protein in liver and skeletal muscle by STAT3 activation.^27^ Moreover, IL-6 circulating levels correlate with cachexia development and poor prognosis in prostate cancer patients.^28^ IL-6 can be produced not only by the immune system but also directly by the tumor,^29^ further highlighting the direct involvement of tumor cells in driving cachexia. Other members of the IL-6 family such as ciliary neurotrophic factor and leukemia inhibitory factor have also been associated with cachexia development.^30, 31^ The upregulation of pro-inflammatory cytokines co-occurs with decreased expression of the cytokines hampering inflammation, such as IL-4 -10 and -12.^32^ Coherently, several treatments controlling excessive inflammation provided beneficial effects on cachexia progression.^32, 33, 34, 35^

### Skeletal muscle wasting

Skeletal muscle represents one of the major compartments of the human body, whose function is necessary for a variety of biological processes, from movement to respiration. A tight balance between protein synthesis and degradation is required to maintain muscle homeostasis^36^ while a decrease in synthesis or an excessive degradation results in wasting.^36^ The complex hormonal network of anabolic and catabolic factors normally regulating this balance^36, 37^ is heavily disrupted during tumor progression.

Indeed, it has been reported that during cachexia, both cancer patients and mouse models, experience a decrease in the circulating levels of the anabolic factor insulin-like growth factor-1 (IGF-1) and the development of insulin resistance.^37, 38, 39, 40, 41, 42, 43^

In parallel with the defective activity of anabolic factors, the production of factors promoting catabolism is augmented both in cachectic mouse models and patients, that is: angiotensin II,^44, 45^ IL-6,^26, 46^ myostatin,^47, 48^ activin A,^49^ interferon gamma and TNFα.^15, 21^ Blockade of activin receptor IIB (ActRIIB), the receptor for several transforming growth factor beta family ligands known to promote atrophy (as activin A and myostatin), was sufficient to reverse cachexia and prevent death in several cancer cachexia mouse models, providing the first formal proof of the direct impact of cachexia on cancer death.^50^ Transforming growth factor beta mechanism of action in promoting cachexia has been elucidated in a recent report. The work from Waning *et al.*^51^ showed that, in several mouse models of bone metastasis, increased transforming growth factor beta signaling (released during osteolysis caused by bone metastasis) promotes skeletal muscle oxidation of the calcium channel RyR1 (ryanodine receptor and calcium release channel), ultimately leading to leaky channels and inefficient muscle activity. Clinical relevance of this finding derives from the fact that also patients with bone metastasis present the same channel oxidation, and that drugs restoring calcium channel functionality prevented cancer-related muscle weakness. Another factor affecting skeletal muscle and upregulated during atrophy is TRAF6 (TNFα receptor adapter protein), which is also overexpressed in muscle from gastric cancer patients.^52, 53^ Its inhibition has been shown to prevent skeletal muscle wasting induced by cachexia in experimental models.^53^

Insulin resistance has been recently modeled in a tumor model of drosophila. In this animal model, insulin signaling was disrupted by the expression of ImpL2, an insulin growth factor binding protein that inhibits both insulin and IGF-1 signaling.^54, 55^ ImpL2 was produced directly by different tumor types, promoting peripheral organs insulin resistance and therefore systemic tissue wasting,^54, 56^ a phenomenon likely present also in cancer patients.^57^ Interestingly, in the drosophila model, insulin/IGF-1 signaling was upregulated in cancer cells, thus allowing to benefit of the systemic hyperglycemia.^55^ Further studies will be required understand the relevance of this mechanism in patients.

At the cellular level, three main degradation pathways have been described in skeletal muscle to account for protein degradation (Table 2), that is, ubiquitin-mediated proteasome degradation (UPR), autophagy and calcium-activated protease calpains.^36, 58, 59^

During tumor cachexia, skeletal muscle specifically upregulates muscle specific UPR system,^60^ in particular by promoting ubiquitin-ligase MurF1 and Atrogin-1 expression.^4, 61^ In a rat model of cachexia, induced by the Yoshida ascites hepatoma, UPR upregulation was evident following tumor growth, as shown by Atrogin-1 messenger RNA^60^ and increased protein ubiquitylation.^60, 62^ The expression of this ubiquitin ligase has been shown to be mainly regulated by the transcription factor FoxO3a (Forkhead Box (Fox) O).^63^ Another transcription factor involved in UPR upregulation is NF-κB, which has been shown to stimulate Murf1 expression following Lewis lung carcinoma (LLC) tumor inoculation and muscle wasting in mice.^64^ NF-κB inhibition with sulfasalazine (in combination with MAPK and proteasome inhibitors) prevented cachexia in a murine model of lung cancer.^65^

Despite the large body of evidence supporting UPR as a major driver of muscle atrophy in murine models, limited evidence are present for this mechanism in human cancer cachexia.^52, 66^

Besides UPR, there is a growing interest in the role of autophagy in mediating skeletal muscle wasting.^67, 68, 69^ Indeed, autophagy has been suggested to be upregulated during cancer cachexia in patients. In a small cohort, lung cancer patients presented increased levels of autophagy mediators BNIP3 (messenger RNA) and LC3B (protein), as well as of the transcription factor promoting autophagy FOXO1.^70^ Similarly, in another study performed on esophageal cancer patients versus weight-stable non-cancerous control patients, autophagy was identified as the main promoter of skeletal muscle proteolysis.^71^ Eventually, in a group of 92 gastrointestinal cancer patients expression of GABARAPL1 (an interactor of lysosomal vesicles and autophagy inducer^72^) was increased compared with healthy controls.^73^

Calpain proteases have been proposed to initiate the degradative process during cachexia,^74^ however, limited information concerning their role in muscle wasting is available.^60^

Adult skeletal muscle normally regenerates after injury through activation and differentiation of a resident population of stem cells called satellite cells. However, the behavior of these cells is deregulated in cachexia, both in murine cancer models and in patients.^75^ NF-κB induces the activation and expansion of the satellite cell pool, but these cells are unable to complete differentiation, thus further worsening the wasting process.^75^

Also, specific tumor-derived micro RNA promoting myoblast and skeletal muscle death have been recently identified in microvesicles,^76^ indicating a direct action of tumor on skeletal muscle. Further studies will be instrumental in defining the specific impact of such structures in mediating the cross-talk between tumor and skeletal muscle.

Cachectic muscle features an impaired mitochondrial metabolism associated with ineffective ATP generation,^77, 78^ dysfunction of the electron transport chain functionality,^79^ lipid alterations in the mitochondrial fraction^78^ and increased expression of mitochondrial uncoupling proteins (UCPs).^80, 81, 82^ UCPs promote proton leak across the inner mitochondrial membrane, therefore, reducing the proton gradient. UCP-1 expression has been shown to disperse proton gradient with concurrent heat generation, while UCP2 and 3 expressions has been proposed as a cellular mean to prevent excessive oxidative stress by inhibiting OXPHOS.^83, 84^

Accordingly, cachectic skeletal muscles and *in vitro* model of cachexia (C2C12-derived myotubes treated with LLC-conditioned medium) present sign of excessive oxidative stress,^79, 85^ responsible for the worsening wasting process, mainly by promoting protein oxidation by reactive oxygen species,^86^ ultimately contributing to muscle weakness.^51^ Mitochondrial dysfunction occurring in the skeletal muscle has been associated with alteration in the lipid content of the mitochondrial fraction, most likely affecting mitochondrial functionality by altering membrane fluidity.^78^

Whether these alterations in UCP levels are actively causing wasting or are an attempt to prevent it, the resulting increase in proton leak might be responsible for the energetic inefficiency typical of this condition; therefore contributing to the overall increase in the resting energy expenditure (REE) normally evident in cachectic patients.^87^ Intriguingly, it has been suggested by *in vitro* experiments that following TNFα treatment, skeletal muscle might promote a futile cycle linked to the co-activation of phosphofructokinase-1 and fructose-1,6-bisphosphatase, resulting in ATP consumption,^88^ further promoting REE.

### Cardiac muscle

The heart is an important target of cachexia. Cardiac alterations are typical in cancer patients^11^ and ultimately results in heart failure and arrhythmia, which are two of the concurring causes of death during cachexia.^6^ Similarly to skeletal muscle, cardiac wasting involves the activation of protein turnover mediated by the UPR system.^89^ Indeed, the heart weight and functionality has been reported to decrease in a murine model of colon cancer^90^ developing chronic heart failure. As in skeletal muscle, NF-κB inhibition has been shown to ameliorate cardiac atrophy and functionality in a mouse model of Colon-26-driven cancer cachexia,^91^ suggesting novel therapeutic approaches for this severe cause of cancer-cachexia death.

Chronic heart failure has been previously associated with the increase of REE,^92^ providing another reason for the increase of energy expenditure in cachectic patients. This increase might be at least in part directly related to an increased metabolism of cardiac tissue, as *ex vivo* hearts from tumor-bearing rats present an increased oxidative rate.^93^

### Liver wasting

One of the main functions of the liver is to act as a biological factory. Indeed, it produces the majority of compound required by the organisms, including glucose, amino acids, fatty acids, cholesterol and hormones regulating several complex functions, such as hepcidin (iron homeostasis), IGF-1 (mass growth), angiotensin (blood pressure) and several coagulation cascade factors. Furthermore, the liver is an organ characterized by high-metabolic rate, substantially contributing to REE.^94^

Albeit neglected (compared with muscle and fat), liver mass substantially increases during cachexia progression,^94, 95^ strongly suggesting the involvement of this organ in cancer cachexia. There is limited evidence concerning the role of liver metabolism on cachexia development. However, liver mass increase in colorectal cancer patients has been shown to correlate with increased energy expenditure,^94^ increased expression of UCPs.^80^ Furthermore, inefficient oxidative phosphorylation, primarily related to an increased mitochondrial cardiolipin accumulation,^96^ has been identified *ex vivo* in liver hepatocytes from a cachectic rat model of peritoneal carcinoma,^97^ indicating a direct involvement of liver tissue in cachexia.

During tumor growth, liver tissue is actively co-opted to perform high-rate gluconeogenesis, using the lactate derived from tumor glycolysis.^98, 99^ This oncological version of the Cori Cycle has been reported by tracing experiments with ^14^C-labeled glucose in metastatic cancer patients in the 70s.^98, 100^

This pathway is extremely energy-demanding since, per each glucose molecule produced, 6 ATP are consumed. The resulting glucose is mostly scavenged by the highly glycolytic tumors; this results in a net negative balance further worsening the higher metabolic rate typical of cancer patients.^101, 102^

Another typical feature of hepatic dysfunction associated with cachexia is the onset of steatosis, present in both patient and murine model.^103, 104^ In fact, it has been shown that tumor-bearing patients or mice injected with the Colon-26 (C26) model are characterized by a rapid decrease in circulating very low-density lipoprotein responsible for the mobilization of lipid in the bloodstream.^105^ From a molecular standpoint, this downregulation has been directly associated with the increased expression of TSC22D4, a transcription factor directly induced by transforming growth factor beta. This inhibits very low-density lipoprotein secretion, lipogenesis and eventually leads to the accumulation of lipids in the liver, which ultimately might promote liver gluconeogenesis.^106^

Although liver contributes to cachexia by increasing energy expenditure through gluconeogenesis and reducing very low-density lipoprotein circulation, it participates as well to the worsening of inflammation by secreting acute phase proteins and reducing albumin secretion, a process mostly driven by IL-6 and TNFα.^107, 108^ This eventually results in muscular protein breakdown and adipocytes lipolysis.^27^

Cancer patients exhibit decreased albumin production, in all likelihood mediated by TNFα, as TNFα treatment *per se* is sufficient to inhibit albumin production *in vivo* (mice and rabbits), or in isolated hepatocytes *in vitro*.^109, 110^ However, a different mechanism has been proposed in pancreatic cancer, where hypoalbuminemia is not associated with decreased synthesis,^111^ but with increased uptake through KRAS-dependent macropinocytosis to sustain energy maintenance.^112, 113^

Altogether, these data indicate that liver directly contributes to cachexia by promoting hypermetabolism and increased energy expenditure. However, further studies are required to evaluate its relative contribution to the cachectic process and to define better the metabolic interplay between tumor and liver.

### Lipid wasting and browning

Albeit not as penetrant as skeletal muscle wasting, adipose tissue depletion has been identified as one of the symptoms of cachexia.^114, 115^ Indeed, cachectic patients manifest high levels of circulating free fatty acids, glycerol and triacylglycerol.^116, 117^ This is linked to the increased circulation of several factors promoting lipid mobilization, such as the adipokine Zn-alpha 2-glycoprotein/lipid-mobilizing factor (ZAG/LMF), IL-1, IL-6 and TNFα.^15, 16, 32^

In specific mouse models of cachexia (LLC lung cancer and B16 melanoma), lipid wasting precedes skeletal muscle loss.^115^ Indeed, preventing triglyceride degradation and lipolysis in mice lacking key lipolytic enzymes (for example, adipose triglyceride lipase and hormone-sensitive lipase) ultimately averts skeletal muscle loss, underlining a close link between these two different tissues.^115^ Interestingly, also cachectic cancer patients present increased triglyceride hydrolase activity.^117^ In addition, the fatty acid mobilization-promoting adipokine ZAG promotes also skeletal muscle protein synthesis and fatty acid oxidation, suggesting that increased level of Zn-alpha 2-glycoprotein during tissue wasting acts as a salvage pathway.^99, 118^

An interesting feature of cancer cachexia is the progressive switch of fat tissue type, from white (white adipose tissue) to brown (brown adipose tissue), which derives its name from the darker color associated with the enrichment in mitochondria.^119, 120^ These mitochondria present high levels of the UCP-1, which directly promotes thermogenesis by uncoupling the electrochemical gradient from ATP generation.^121^ Browning strongly contributes to the increased energy expenditure common in cachectic patients.^119^ Pro-inflammatory factors either derived from the host immune system or the tumor, contribute to this switch.^119, 120^ In LLC tumor-bearing mice, several tumor-derived cytokines correlate with the induction of tissue browning and, therefore, with increased energy expenditure.^119^ In particular, cachectic lipid wasting occurs mostly in tumors actively secreting parathyroid hormone-related protein.^119^ This hormone is augmented as well in several cancer patients and has been previously associated with hypercalcemia, a common metabolic abnormality in many cancer types.^122^ Collectively, fat tissue wasting can be interpreted as a critical turning point in the cachectic process, as it further contributes to the propagation of cachexia by stimulating skeletal muscle wasting.^115^ As it is also emerging that tumors require fatty acid oxidation,^123^ it will be important to assess the impact of lipolysis on tumor progression.

### Brain and food intake

Decreased in appetite and alterations in taste perception are common features in cancer patients.^11, 124^ In particular, cancer anorexia is a characteristic of end-stage patients contributing to the worsening of cachexia. Although this is in part related to the development of depressive disorders associated with the psychological implications of having cancer,^8^ it is also bound to the alterations in the complex hormonal network regulating appetite.^10^ A pivotal player in cachexia development is the hypothalamus, which regulates both food intake and body energy expenditure.^125^ Several factors regulates food intake, including the vagal stimulation induced by gastric distension bound to food intake^126, 127^ and hormones. Hormones produced peripherally either promote food intake (orexigenic, like ghrelin), or inhibit it (anorexigenic, such as leptin, insulin, cholecystokinin, peptide YY and glucagon-like peptide 1/GLP1).^128^ At central level in the hypothalamus, peripheral signals regulates the axis promoting food intake such as the Neuropeptide Y (NPY) and Agouti-related protein (AgRP) or the anorexigenic one, such as pro-opiomelanocortin (POMC) precursor related to the production of melanocyte-stimulating hormone α-MSH.^129^ Despite the peripheral signals triggering food intake are maintained in some cachectic conditions, it has been observed a decreased responsiveness of the hypothalamus.^130^ This is the case for ghrelin, whose levels are normally increased in cachectic patients, but without food intake promotion,^42^ a phenomenon named ‘ghrelin resistance'.^42, 131^

In addition, in cancer it has been reported an increased resistance to the hormones promoting food intake, such as Neuropeptide Y and Agouti-related protein.^132, 133^

On the contrary, an increased activation of pro-anorexigenic factors derived by hypothalamic melanocortin system has been shown.^134, 135^

Tumor-associated inflammation is involved in this process (or these processes) as several pro-inflammatory cytokines such as TNFα, interferon gamma, IL-1 and IL-6, directly promote these alterations^136^ and, coherently, anti-inflammatory drugs such as cyclooxygenase inhibitors ameliorates cancer anorexia.^137, 138^

In addition, TNFα has also been associated to the stimulation of bitterness perception,^139^ thus further inhibiting the willingness of food assumption.

Altogether these cytokines are responsible for the so-called ‘sickness behavior', a common disease state typical of many chronic disease and proposed as an evolutionary response involved in fighting infections by depleting iron and nutrients required for bacterial growth.^140^

The direct involvement of tumor-derived factors in promoting anorexia has been proposed.^141, 142^ Coherently, the bioactive lipid sphingosine-1-phosphate, which is involved in cancer progression and is produced by several tumors,^143^ has been recently shown to promote energy expenditure and anorexia.^135^ Increased engagement of sphingosine-1-phosphate-receptor on hypothalamic neurons promotes increased temperature and oxygen consumption, while decreasing food intake.^135^

In fact, hypothalamus controls a wide range of biological activities, including energy expenditure regulation and glucose homeostasis.^144^ Coherently, during cachexia hypothalamic activity specifically mediates increased energy expenditure,^135, 145^ it will be important, therefore, to define the role of hypothalamus in regulating glucose homeostasis.

Appetite improvement have been achieved in cancer patients with the progesterone analog megestrol acetate (by an unclear mechanism)^146^ and by the ghrelin analog anamorelin,^41^ thus providing novel therapeutic avenues in the treatment of such complication.

### Pancreas

The identification of decreased glucose tolerance in cancer patients, dating back to 1919, identified glucose metabolism as the first metabolic abnormality in cancer.^147^ Although insulin resistance is a strong risk factor for cancer development,^148, 149^ tumor progression can promote insulin resistance by itself. Indeed, it has been shown that several cancer patients present insulin resistance^150, 151^ that progressively worsen during cachexia development.^10^ Furthermore, the degree of glucose tolerance positively correlates with mortality risk.^152^

Decreased insulin sensitivity during tumor progression has also been reported in drosophila,^54, 55^ and in mice,^39^ where Colon-26 tumor inoculation was sufficient to induce insulin resistance before cachexia onset,^39^ while in Walker 256 tumor-bearing rats isolated Langerhans islet were more resistant to glucose challenge, resulting in decreased insulin secretion.^153^

One of the factors identified for the induction of insulin resistance is TNF-α, which directly impairs insulin signaling and IRS-1 activation.^154^

Insulin has several metabolic activities that can affect tumor progression.^155, 156^ The increase in insulin level, as it happens in the onset of insulin resistance, *per se* promotes directly tumor growth by acting as growth factors. Coherently, several tumors overexpress the insulin receptor and IGF-1 receptor.^157, 158^ However, insulin resistance can also promote tumor growth indirectly by modulating host metabolism in at least two independent manners. On one side, as insulin is an anabolic factor that normally blocks protein breakdown and promotes protein synthesis;^159, 160^ insulin resistance (and similarly IGF-1-resistance) might promote muscle wasting, hence, amino acid mobilization into the circulation, potentially fueling cancer. On the other, insulin signaling impairment also promotes liver gluconeogenesis,^161^ further increasing REE, tissue wasting and ultimately fueling cancer aerobic glycolysis.

The role of insulin signaling in preventing cachexia is further stressed by experimental data showing that mice treated with insulin sensitizers (rosiglitazone)^39, 162^ and patients treated with insulin ameliorate cachexia symptoms.^163^

Not only insulin secretion is affected by tumor progression, but also glucagon levels are increased.^164^ The increased production of glucagon in the alpha islet of pancreas during cancer progression further promotes liver gluconeogenesis as reported in different tumor models, both in humans and in animal models.^164, 165, 166^

Albeit there is no clear mechanism behind the induction of this hormone by the tumor, normalizing its levels has been suggested to impair cachexia progression.^166^

### Gastrointestinal tract

Gut functionality contributes to cachexia. This is particularly relevant in gastrointestinal tumors, as proven in a mouse model of colon cancer (transgenic APC^+/min^ strain), where gut barrier was disrupted along with tumor growth, resulting in increased systemic inflammation and endotoxemia.^167, 168^

Other than colon cancers, a broader impact of gut on cachexia is bound to gut microbiota.^169^ The human body is in symbiosis with the gut microbiota, which identify the portion of microorganism residing in the intestinal tract outnumbering human cells by a 10-fold factor.^170^ Alteration of the gut flora due to undernutrition and chemotherapy ultimately affects specific metabolite availability and absorption,^169, 171^ which in turn affects tumor growth and cachexia.^172, 173^ The gastrointestinal tract, and mainly the stomach, is also the source of the orexigenic peptide ghrelin, which is strongly increased during cachexia.^42^ As ghrelin exerts several other activities, ranging from increasing adiposity,^174^ reducing REE^175^ and impairing muscle atrophy,^176^ it is possible that its expression is induced as a compensatory mechanism to buffer cachexia. Owing to the pleiotropic effects of ghrelin (partly shared with its unacylated form through an unknown receptor^176, 177, 178^), the use of ghrelin analog, Anamorelin, has a strong therapeutic potential. Indeed, early clinical trials are suggesting beneficial effect of Anamorelin in improving both appetite and skeletal muscle mass.^41, 179^

### Cachexia: a cancer target or an innocent bystander?

As cachexia associates with several pathological conditions, such as chronic inflammation, cardiac disease and AIDS,^37^ cancer cachexia has always been regarded as an epiphenomenon of tumor progression,^6^ and even its role on death promotion has only been formally proven recently.^50^

**Figure 1:**
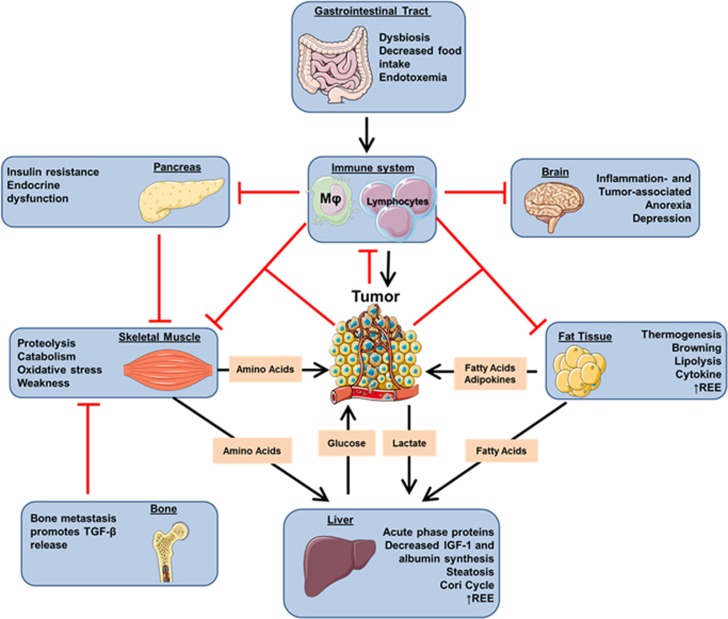
The simplified scheme represents the major organs commonly affected during cachexia progression and how they fuel tumor growth. In brief, tumor tissue and the co-opted immune system secrete specific factors, thus promoting skeletal muscle wasting and lipolysis. Pro-inflammatory cytokines contribute to develop anorexia and insulin resistance, ultimately worsening skeletal muscle wasting. Gastrointestinal tract tumors and bone metastasis can promote further cachexia by causing endotoxemia and transforming growth factor beta release, respectively. (Adapted from Servier Medical Art, www.servier.com).

However, mounting evidence supports the notion that cachexia is not only a severe complication of tumor growth, but results from the systemic metabolic reprogramming of the host to grow and progress^180^ (Figure 1).

In fact, tumor can promote cachexia by secreting tissue-wasting factors^10, 76, 87, 119, 120^ and by promoting dysfunction in specific organs, such as liver,^105^ gut, immune system, brain and pancreas.

Interesting data concerning the active interplay between tumor and cachexia derive from the study of pancreatic ductal adenocarcinoma, where cachexia development occurs in almost 90% of the cases.^1^ In this tumor type, increased amino acid levels are an early marker of disease occurrence^181^ in patients, years before pancreatic ductal adenocarcinoma diagnosis. Coherently, in a K-RAS-driven transgenic model of pancreatic cancer the increase in circulating amino acid was present before the actual tumor was detectable.^181^ This effect on the host, even at early phase of tumor growth, when no evidence of discomfort is present, indicates the importance of actively understanding the interplay between tumor and the host on a systemic level, with a particular focus toward the metabolic dependencies of growing tumors. Further studies will be required to evaluate if this phenomenon is specific to pancreatic ductal adenocarcinoma or if it is a feature of different tumor types.

As tumor is a highly energy-demanding tissue, energy and metabolic intermediates are required to sustain proliferation and cell-death resistance,^182^ it is not surprising, therefore, that tumor cells promote metabolic reprogramming not only cell-autonomously, but of the whole organism.

Although the study of cancer metabolism has been mostly focused toward the definition of aerobic glycolysis, several other metabolic pathways are emerging as important for tumor development. Because of the inefficient perfusion typical of cancers^183^ along with the avidity of cancer cells for glucose,^184^ other carbon sources are required for tumor growth.^185^

For instance, several studies indicate that specific metabolite uptake is required for tumor growth and progression in some cases, such as lipids,^186, 187^ branched amino acids,^188^ glutamine,^189, 190^ serine^191^ or even entire proteins by macropinocytosis.^112, 113^ By actively scavenging different nutrients from the bloodstream, cancer cells manage severe nutrient deprivation.^113^ Altogether, tumor cells have a metabolic advantage from the induction of systemic tissue wasting, prompting to reconsider cachexia as a part of the metabolic program in tumor development. A further indication for this mechanism derives from the work of Luo *et al.*^192^ who identified a metabolic cross-talk occurring in colorectal cancers between tumor and skeletal muscle. To aim this, colon cancer cells release high-motility group box 1 (HMGB1), which contributes to the metabolic reprogramming of skeletal muscle through RAGE (Receptor for Advanced Glycation End-products), inducing autophagy and release of free amino acids in the plasma. This results in the transfer of carbon skeleton from the muscle to the tumor as demonstrated by ^13^C-glutamine tracing.^192^ Considering that high-motility group box 1 circulating levels correlate with the severity in different cancer types,^193, 194, 195^ it will be important to explore this cross-talk in different tumor types.

Cancer cachexia it is not merely a complication of tumor progression, as cancer cells induce and exploit systemic functions. It would be reductive to consider cachexia as solely caused by tumor metabolism acting as ‘energy sink' as initially proposed,^1, 196^ as evidences by the fact that tumor mass hardly correspond to the severity of cachexia.^10, 105^ Indeed, it is a syndrome induced also by several other noxious factor, from chronic infections to cardiac or respiratory failure.^1^

## Concluding remarks

As cancer cachexia is being progressively defined, also from a molecular standpoint, it will be important to discriminate between the alterations actually promoting tumor progression and the one which are simple off-targets of the abnormal levels of factors presents during tumor growth. Understanding the effects of tumor on the entire organism and identify the signaling pathways involved will allow more effective cancer therapies and, ultimately, a better quality of life for patients.

Although several molecular mechanisms driving cachexia have been identified by using murine models, it will be vital to define the real impact of such processes in human patients. Moreover, relatively few murine models are used to generate cachexia (the vast majority being LLC in C57BL/6, and Colon-26 in Balb/c, tumor cells injection), while the use of transgenic mice is generally restricted to C57BL/6 APC^+/min^.^26^ The use of different strains and different cancer types will be essential to model the variety of the cachectic processes occurring in cancer patients, especially considering that cachexia has different penetrance according to the associated cancer type.^10^

Further efforts will be required to define the pathogenesis of cachexia in patients at early stages (that is, the pre-cachectic stage^197^), where systemic alterations are more likely to be reversible. As cancer cachexia affects different tissue at the same time, it will also be pivotal to devise therapeutic strategies with multiple targets.

## Figures and Tables

**Table 1:** Cytokines mainly associated with the pathogenesis of cachexia, evidence derived from human studies is italicized

*Pro-inflammatory cytokines*			
TNFα		Promotes tissue proteolysis and NF-kB activation	Han *et al.*^18^
		Promotes anorexia and fatigue in cancer patients	Jakubowski et al.^17^
IL-1		Promotes anorexia	Uehara *et al.*^23^
		Genetic polymorphisms resulting in increased IL-1β levels are marker of poor prognosis	Graziano et al.^25^
IL-6		Increased circulating levels are poor prognosis markers	Kuroda et al.^28^ Mantovani et al.^46^
		It can be produced directly by the tumor and trigger cachexia	Baltgalvis *et al.*^26^
		Increased fat tissue browning	Petruzzelli *et al.*^120^
IFNγ		Synergize with TNFα in promoting muscle wasting	Acharyya *et al.*^21^

Abbreviations: IFNγ, interferon gamma; IL-1, interleukin-1; IL-6, interleukin-6; TNFα, tumor necrosis factor alpha.

**Table 2:** Molecular mechanisms driving skeletal muscle atrophy during cachexia, evidence derived from human studies are *italicized*

*Skeletal muscle wasting*		
UPR	Upregulation of the ubiquitin-proteasome pathway in cancer model	Baracos *et al.*^60^
	Proteasome and NF-kB inhibitors prevent experimental cancer cachexia	Chacon-Cabrera *et al.*^65^
	UPR activation is required for muscle atrophy	Bodine *et al.*^59^
Authophagy	It is induced in the skeletal muscle of cancer patients	Op den Kamp et al.^70^ Tardif et al.^71^ Boyer-Guittaut et al.^72^
	Promotes muscle wasting during cachexia	Penna *et al.*^68^
ActRIIB	Decoy receptor reverses muscle wasting	Zhou *et al.*^50^
	Cachectic patients present increased circulating levels of ActRIIB ligand, activin	Loumaye et al.^49^
	Myostatin (ActRIIB ligand) knock-out prevents experimental cachexia	Gallot *et al.*^48^
		
*Lipid wasting*		
Lipolysis	Adipose Triglyceride Lipase inhibition prevents muscle wasting in experimental cachexia.	Das and Hoefler^117^
	Cachectic cancer patients present increased lipolytic activity	

Abbreviations: ActRIIB, activin receptor IIB; UPR, ubiquitin-mediated proteasome degradation.

## Abbreviations

IGF-1: insulin-like growth factor-1
IL-: interleukin-
LLC: lewis lung carcinoma
REE: resting energy expenditure
TNFα: tumor necrosis factor alpha
UCPs: uncoupling proteins
UPR: Ubiquitin proteasome degradation.
